# A two-point-Padé-approximant-based method for bounding some trigonometric functions

**DOI:** 10.1186/s13660-018-1726-7

**Published:** 2018-06-20

**Authors:** Xiao-Diao Chen, Junyi Ma, Jiapei Jin, Yigang Wang

**Affiliations:** 1 Key Laboratory of Complex Systems Modeling and Simulation, Hangzhou Dianzi University, Hangzhou, China; 2 School of Media and Design, Hangzhou Dianzi University, Hangzhou, China

**Keywords:** Wilker’s inequality, Trigonometric approximation, Padé approximant, Two-sided bounds, Becker–Stark’s inequality

## Abstract

Inequalities are frequently used for solving practical engineering problem. There are two key issues of bounding inequalities; one is to find the bounds, and the other is to prove the bounds. This paper takes Wilker type inequalities as an example, presents a two-point-Padé-approximant-based method for finding the bounds, and it also provides a method to prove the bounds in a new way. It not only recovers the estimates in Mortici’s method, but it also provides new improvements of estimates obtained from prevailing methods. In principle, it can be applied for other inequalities.

## Introduction

The Wilker inequality, which involves the trigonometric function

1 $$ f(x)= \biggl(\frac{\sin x}{x} \biggr)^{2} + \frac{\tan x}{x}, $$

has been discussed in the recent past; see also [2, 3, 6–9, 11–15, 17–23] and the references therein, such as the following ones in [14, 18]:

2 $$\begin{aligned} &2+\frac{16}{\pi^{4}} x^{3} \tan x < f(x) < 2+ \frac{8}{45} x^{3} \tan x, \quad 0< x< \pi/2, \end{aligned}$$

3 $$\begin{aligned} &2+ \biggl(\frac{8}{45}-a(x) \biggr) x^{3} \tan x < f(x) < 2+ \biggl(\frac{8}{45}-b(x) \biggr) x^{3} \tan x, \quad 0< x< 1, \end{aligned}$$

4 $$\begin{aligned} &2+ \biggl(\frac{16}{\pi^{4}} + c(x) \biggr) x^{3} \tan x < f(x), \quad ( \pi-1)/2 < x < \pi/2, \end{aligned}$$

5 $$\begin{aligned} &f(x) < 2+ \biggl(\frac{16}{\pi^{4}}+d(x) \biggr) x^{3} \tan x, \quad \pi/3-1/2 < x < \pi/2, \end{aligned}$$

where $a(x)=\frac{8}{945} x^{2}$, $b(x)=\frac{8}{945} x^{2}-\frac{16}{14\mbox{,}175} x^{4}$, $c(x)=(\frac{160}{\pi^{5}}-\frac{16}{\pi^{3}})(\frac{\pi}{2}-x)$, $d(x)=(\frac{160}{\pi^{5}}-\frac{16}{\pi^{3}})(\frac{\pi}{2}-x)+(\frac{960}{\pi^{6}}-\frac{96}{\pi^{4}})(\frac{\pi}{2}-x)^{2}$.

Recently, Nenezić, Malešević and Mortici provided inequalities within the extended interval $(0,\pi/2)$ [15], e.g., Eq. (7) extends both Eq. (4) and Eq. (5), while Eq. (6) extends the left side of Eq. (3). We have

6 $$\begin{aligned} &2+ \biggl(\frac{8}{45}-a(x) \biggr) x^{3} \tan x < f(x) < 2+ \biggl( \frac{8}{45}-b_{1}(x) \biggr) x^{3} \tan x,\quad 0< x< \pi/2, \end{aligned}$$

7 $$\begin{aligned} &2+ \biggl(\frac{16}{\pi^{4}} + c(x) \biggr) x^{3} \tan x < f(x)< 2+ \biggl(\frac{16}{\pi^{4}}+d(x) \biggr) x^{3} \tan x,\quad 0< x < \pi/2, \end{aligned}$$

where $b_{1}(x)=\frac{8}{945} x^{2}-\frac{\alpha}{14\mbox{,}175} x^{4}$ with $\alpha = \frac{480 \pi^{6} - 40\mbox{,}320 \pi^{4} + 3\mbox{,}628\mbox{,}800}{\pi^{8}} \approx 17.15041$.

In this paper, we consider

8 $$ F(x)=f(x) \cdot \cos(x) = \biggl(\frac{\sin x}{x} \biggr)^{2} \cdot \cos(x) + \frac{\sin x}{x} $$

instead of $f(x)$, which is bounded for $x \in (0,\pi/2]$. Firstly, we present a two-point-Padé approximant-based method [1] to find the two bounding functions

9 $$ L(x)=l_{1}(x) \cdot \cos(x) + l_{2}(x)\cdot \sin(x),~ R(x)=r_{1}(x) \cdot \cos(x) + r_{2}(x)\cdot \sin(x), $$

such that

10 $$ L(x) \leq F(x) \leq R(x), \quad 0 \leq x \leq \pi/2, $$

where $l_{i}(x)$ and $r_{i}(x)$ are unknown polynomials to be determined. Note that $\cos(x)>0, \forall x \in (0,\pi/2)$, from Eq. (10), we obtain

11 $$ l_{1}(x) + l_{2}(x)\cdot \tan(x) \leq f(x) \leq r_{1}(x) + r_{2}(x)\cdot \tan(x), \quad 0 \leq x \leq \pi/2. $$

Secondly, we also provide a new way for proving it.

## The two-point-Padé approximant-based method and examples

Given an interval $[a,b] \subseteq [0,\pi/2]$. From Eq. (9), let

12 $$ l_{i}(x)=\sum_{j=0}^{p_{i}} \alpha_{i,j} x^{j} \quad \mbox{and}\quad r_{i}(x)= \sum_{j=0}^{q_{i}} \beta_{i,j} x^{j}, $$

where $p_{i}, q_{i} \geq 2$, $\alpha_{i,j}$ and $\beta_{i,j}$ are the unknowns to be determined, and $i=1,2$; so there are $n_{p}=p_{1}+p_{2}+2$ and $n_{q}=q_{1}+q_{2}+2$ unknowns in $L(x)$ and $R(x)$ in Eq. (9), respectively. Let $E_{1}(x)=F(x)-L(x)$ and $E_{2}(x)=F(x)-R(x)$. For the sake of convenience, we introduce Theorem 3.5.1 in Page 67, Chap. 3.5 of [4] as follows.

### Theorem 1

*Let* $w_{0}, w_{1}, \ldots, w_{r}$ *be* $r+1$ *distinct points in* $[a,b]$, *and* $n_{0}, \ldots, n_{r}$ *be* $r+1$ *integers* ≥0. *Let* $N=n_{0}+ \cdots + n_{r} + r$. *Suppose that* $g(t)$ *is a polynomial of degree* *N* *such that* $g^{(i)}(w_{j})=f^{(i)}(w_{j})$, $i=0,\ldots, n_{j}$, $j=0,\ldots, r$. *Then there exists* $\xi_{1}(t) \in [a,b]$ *such that* $f(t)-g(t)=\frac{f^{(N+1)}(\xi_{1}(t))}{(N+1)!} \prod^{r}_{i=0} (t-w_{i})^{n_{i}+1}$.

We introduce the following constraints:

13 $$ \textstyle\begin{cases} E_{1}^{(i)}(a)=0, \qquad E_{1}^{(j)}(b)=0,~i=0,1,\ldots,k, \quad \mbox{and }j=0,1,\ldots,N_{1}, \\ E_{2}^{(i)}(a)=0, \qquad E_{2}^{(j)}(b)=0,~i=0,1,\ldots,l, \quad \mbox{and }j=0,1,\ldots,N_{2}, \end{cases} $$

where $N_{1} \geq n_{p}-k-1$ and $N_{2} \geq n_{q}-l-1$. By selecting suitable *k* and $N_{1}$, we can find $n_{p}$ constraints for determining $L(x)$; similarly, by selecting suitable *l* and $N_{2}$, we can find $n_{q}$ constraints for determining $R(x)$. Combining Theorem 1 with Eq. (13), there exists $\xi_{i}(x) \in [a,b], i=1,2$, such that

14 $$ \textstyle\begin{cases} E_{1}(x)=\frac{E_{1}^{(N_{1}+k+2)}(\xi_{1}(x))}{(N_{1}+k+2)!} (x-a)^{k+1} (x-b)^{N_{1}+1}, \quad x \in [a,b], \\ E_{2}(x)=\frac{E_{2}^{(N_{2}+l+2)}(\xi_{2}(x))}{(N_{2}+l+2)!} (x-a)^{l+1} (x-b)^{N_{2}+1}, \quad x \in [a,b]. \end{cases} $$

From Eq. (14), if $(-1)^{d} \cdot E_{1}^{(N_{1}+k+2)}(\xi_{1}(x)) \geq 0$, $\forall x \in [a,b]$, we have $E_{1}(x) \cdot (-1)^{N_{1}+1+d} \geq 0$, where $d=0$ or $d=1$; similarly, if $(-1)^{d} \cdot E_{2}^{(N_{2}+l+2)}(\xi_{2}(x)) \geq 0$, $\forall x \in [a,b]$, we have $E_{2}(x) \cdot (-1)^{N_{2}+1+d} \geq 0$. Based on the above observations, one may find the bounding functions in the above way.

We will show three examples which recover or refine previous Wilker type inequalities, including Eq. (2), Eq. (6) and Eq. (7), where $c_{j}$ is a unknown coefficient to be determined by interpolation constraints.

### Example 1

Let $L_{1}(x)=2 \cos(x)+ c_{1} \sin(x)$ and $R_{1}(x) = 2 \cos(x) + c_{2} \sin(x)$, $E_{1,l}(x)=F(x)-L_{1}(x)$ and $E_{1,r}(x)=F(x)-R_{1}(x)$, $x \in [0,\pi/2]$. It can be verified that $E_{1,i}^{(j)}(0)=0$, where $j=0,1,2,3$, $i=l,r$. By applying the constraints $L_{1}(\pi/2)=F(\pi/2)$ and $R_{1}^{(4)}(0)=F^{(4)}(0)$, we obtain $c_{1}=\frac{16}{\pi^{4}}$ and $c_{2}=\frac{8}{45}$, respectively, which recovers Eq. (2).

### Example 2

Let $L_{2}(x)=2 \cos(x)+ (c_{3}+c_{4} x + c_{5} x^{2}) x^{3} \sin(x)$ and $R_{2}(x) = 2 \cos(x) + (c_{6}+c_{7} x^{2} +c_{8} x^{4}) x^{3} \sin(x)$, $E_{2,l}(x)=F(x)-L_{2}(x)$ and $E_{2,r}(x)=F(x)-R_{2}(x)$, $x \in [0,\pi/2]$. It can be verified that $E_{2,i}^{(j)}(0)=0$, where $j=0,1,2,3$, $i=l,r$. By applying the constraints $L_{2}^{(j)}(0)=F^{(j)}(0)$, $j=4,5,6$, we obtain $c_{3} = \frac{8}{45}$, $c_{4}=0$ and $c_{5}=-\frac{8}{945}$, which recovers the left side of Eq. (6). By applying the constraints $R_{2}^{(4)}(0)=F^{(4)}(0)$, $R_{2}^{(5)}(0)=F^{(5)}(0)$ and $R_{2}(\pi/2)=F(\pi/2)$, we obtain $c_{6} = \frac{8}{45}$, $c_{7}=-\frac{8}{945}$ and $c_{8}=\frac{\alpha}{14\mbox{,}175}$, which recovers the right side of Eq. (6).

### Example 3

Let $L_{3}(x)=2 \cos(x)+ (c_{9}+c_{10} (\pi/2-x) ) x^{3} \sin(x)$, $R_{3}(x) = 2 \cos(x) + (c_{11}+c_{12} (\pi/2-x) + c_{13}(x-\pi/2)^{2}) x^{3} \sin(x)$, $E_{3,l}(x) = F(x)-L_{3}(x)$ and $E_{3,r}(x) = F(x)-R_{3}(x)$, $x \in [0,\pi/2]$. It can be verified that $E_{3,i}^{(j)}(0)=0$, where $j=0,1,2,3$, $i=l,r$. By applying the constraints $L_{3}(\pi/2)=F(\pi/2)$ and $L_{3}'(\pi/2)=F'(\pi/2)$, we obtain $c_{9} = \frac{16}{\pi^{4}}$ and $c_{10}=\frac{160}{\pi^{5}}-\frac{16}{\pi^{3}}$, which recovers the left side of Eq. (7). By applying the constraints $R_{3}^{(j)}(\pi/2)=F^{(j)}(\pi/2)$, $j=0,1,2$, we obtain $c_{11} = \frac{16}{\pi^{4}}$, $c_{12}=\frac{160}{\pi^{5}}-\frac{16}{\pi^{3}}$ and $c_{13}=\frac{960}{\pi^{6}}-\frac{96}{\pi^{4}}$, which recovers the right side of Eq. (7).

## Results

This section finds other two bounding functions $L(x)$ and $R(x)$ to improve the bounds of Eq. (6) and Eq. (7). Combining Eq. (12) with Eq. (13), by setting $p_{1}=q_{1}=4$, $p_{2}=q_{2}=5$, $k=8$, $N_{1}=1$, $l=7$ and $N_{2}=2$, we obtain $L(x)$ and $R(x)$ in Eq. (10) as

$$\begin{aligned} &L(x)=l_{1}(x) \cdot \cos(x)+ l_{2}(x) \cdot \sin(x) = \Biggl(\sum_{j=0}^{4} {\alpha_{1,j} x^{j}} \Biggr) \cdot \cos(x)+ \Biggl(\sum_{j=0}^{5} {\alpha_{2,j} x^{j}} \Biggr) \cdot \sin(x), \\ &R(x)= r_{1}(x) \cdot \cos(x)+ r_{2}(x) \cdot \sin(x) = \Biggl(\sum_{j=0}^{4} {\beta_{1,j} x^{j}} \Biggr)\cdot \cos(x) + \Biggl(\sum_{j=0}^{5} {\beta_{2,j} x^{j}} \Biggr) \cdot \sin(x), \end{aligned}$$

where

$$\begin{aligned} &\lambda_{1}=\frac{16 (2 \pi^{10}-177 \pi^{8}+4935 \pi^{6}-85\mbox{,}050 \pi^{4}+831\mbox{,}600 \pi^{2}-3\mbox{,}175\mbox{,}200)}{(\pi^{8}-360 \pi^{6}+35\mbox{,}760 \pi^{4}-604\mbox{,}800 \pi^{2}+2\mbox{,}822\mbox{,}400) \pi}, \\ & \alpha_{1,1}=\frac{ \lambda_{1}}{3},\qquad \alpha_{1,3}= \frac{2\lambda_{1}}{-63},\qquad \alpha_{2,2}=\frac{\lambda_{1}}{7}, \\ &\lambda_{2}=\frac{8(3 \pi^{10}-308 \pi^{8}+9300 \pi^{6}-132\mbox{,}720 \pi^{4}+957\mbox{,}600 \pi^{2}-2\mbox{,}822\mbox{,}400)}{(\pi^{8}-360 \pi^{6}+35\mbox{,}760 \pi^{4}-604\mbox{,}800 \pi^{2}+2\mbox{,}822\mbox{,}400) \pi^{2}}, \\ & \alpha_{1,2}= - \lambda_{2}, \qquad \alpha_{2,1}= \lambda_{2}, \\ & \lambda_{3} = \frac{(11 \pi^{10}-1065 \pi^{8}+25\mbox{,}935 \pi^{6}-346\mbox{,}500 \pi^{4}+2\mbox{,}885\mbox{,}400 \pi^{2}-10\mbox{,}584\mbox{,}000)}{(\pi^{8}-360 \pi^{6}+35\mbox{,}760 \pi^{4}-604\mbox{,}800 \pi^{2}+2\mbox{,}822\mbox{,}400) \pi^{2}}, \\ &\alpha_{1,4}=\frac{64 \lambda_{3}}{315},\qquad \alpha_{2,3}= \frac{32 \lambda_{3}}{-35}, \qquad \alpha_{1,0}=2, \qquad \alpha_{2,0}=- \alpha_{1,1},\qquad \alpha_{2,4}=\frac{16 \lambda_{1}}{-315}, \\ &\alpha_{2,5}= \frac{32(2 \pi^{10}-141 \pi^{8}-1965 \pi^{6}+51\mbox{,}660 \pi^{4}+12\mbox{,}600 \pi^{2}-2\mbox{,}116\mbox{,}800)}{315(\pi^{8}-360 \pi^{6}+35\mbox{,}760 \pi^{4}-604\mbox{,}800 \pi^{2}+2\mbox{,}822\mbox{,}400) \pi^{2}}; \\ & \lambda_{4}=\frac{16(7 \pi^{10}-90 \pi^{8}-2445 \pi^{6}+94\mbox{,}500 \pi^{4}-1\mbox{,}134\mbox{,}000 \pi^{2}+4\mbox{,}536\mbox{,}000)}{(5 \pi^{10}-558 \pi^{8}+12\mbox{,}480 \pi^{6}-177\mbox{,}120 \pi^{4}+1\mbox{,}756\mbox{,}800 \pi^{2}-7\mbox{,}257\mbox{,}600) \pi}, \\ & \beta_{1,1}= \lambda_{4},\qquad \beta_{2,0}=- \lambda_{4},\qquad \beta_{1,3}=\frac{2 \lambda_{4}}{-21},\qquad \beta_{1,0}=2, \\ & \beta_{1,2}=\frac{40(\pi^{12}-234 \pi^{10}+6180 \pi^{8}-8568 \pi^{6}-1\mbox{,}572\mbox{,}480 \pi^{4}+20\mbox{,}260\mbox{,}800 \pi^{2}-76\mbox{,}204\mbox{,}800)}{21(5 \pi^{10}-558 \pi^{8}+12\mbox{,}480 \pi^{6}-177\mbox{,}120 \pi^{4}+ 1\mbox{,}756\mbox{,}800 \pi^{2}-7\mbox{,}257\mbox{,}600) \pi^{2}}, \\ &\beta_{2,1}=- \beta_{1,2}, \\ & \beta_{1,4}= \frac{32(12 \pi^{12}+4615 \pi^{10}-188\mbox{,}175 \pi^{8}+2\mbox{,}650\mbox{,}200 \pi^{6}-11\mbox{,}692\mbox{,}800 \pi^{4}-45\mbox{,}360\mbox{,}000 \pi^{2}+381\mbox{,}024\mbox{,}000)}{105(5 \pi^{10}-558 \pi^{8}+12\mbox{,}480 \pi^{6}-177\mbox{,}120 \pi^{4}+1\mbox{,}756\mbox{,}800 \pi^{2}-7\mbox{,}257\mbox{,}600) \pi^{4}}, \\ &\beta_{2,2}= \frac{3 \lambda_{4}}{7}, \\ & \beta_{2,3}=\frac{32(\pi^{14}-165 \pi^{12}+3108 \pi^{10}+13\mbox{,}401 \pi^{8}-980\mbox{,}280 \pi^{6}+9\mbox{,}933\mbox{,}840 \pi^{4}-22\mbox{,}680\mbox{,}000 \pi^{2}-76\mbox{,}204\mbox{,}800)}{21(5 \pi^{10}-558 \pi^{8}+12\mbox{,}480 \pi^{6}-177\mbox{,}120 \pi^{4}+1\mbox{,}756\mbox{,}800 \pi^{2}-7\mbox{,}257\mbox{,}600) \pi^{4}}, \\ & \beta_{2,4}=\frac{16 \lambda_{4}}{-105}, \\ &\beta_{2,5}= \frac{32(13 \pi^{12}-2050 \pi^{10}+58\mbox{,}995 \pi^{8}-616\mbox{,}200 \pi^{6}+882\mbox{,}000 \pi^{4}+25\mbox{,}704\mbox{,}000 \pi^{2}-127\mbox{,}008\mbox{,}000)}{-105(5 \pi^{10}-558 \pi^{8}+12\mbox{,}480 \pi^{6}-177\mbox{,}120 \pi^{4}+1\mbox{,}756\mbox{,}800 \pi^{2}-7\mbox{,}257\mbox{,}600) \pi^{4}}. \end{aligned}$$

In principle, more bounds can be found by setting different parameters in Eq. (12) and Eq. (13). The main result is as follows.

### Theorem 2

*We have* $L(x) \leq F(x) \leq R(x)$, $\forall x \in [0,\pi/2]$.

### Proof

(1) Firstly, we give the bounds of $\sin(x)$, $\cos(x)$ and $\sin(2x)$. Let $\Delta_{1,1}(x)=\sin(x)-P_{1}(x)$, $\Delta_{1,2}(x)=\sin(x)-Q_{1}(x)$, $\Delta_{2,1}(x)= \cos(x) - P_{2}(x)$, $\Delta_{2,2}(x)= \cos(x) - Q_{2}(x)$, $\Delta_{3,1}(x)= \sin(2x)/2-P_{3}(x)$, $\Delta_{3,2}(x)= \sin(2x)/2-Q_{3}(x)$, where $P_{1}(x)$, $Q_{1}(x)$, $P_{2}(x)$, $Q_{2}(x)$, $P_{3}(x)$ and $Q_{3}(x)$ are polynomials of degree 12, 12, 13, 13, 15 and 15, respectively. By introducing the following constraints:

15 $$ \begin{aligned} &\Delta_{1,1}^{(i)}(0)=0,\qquad \Delta_{1,1}^{(j)}(\pi/2)=0, \quad i=0,1,\ldots,10, j=0,1; \\ &\Delta_{1,2}^{(i)}(0)=0,\qquad \Delta_{1,2}^{(j)}( \pi/2)=0, \quad i=0,1,\ldots,9, j=0,1,2; \\ &\Delta_{2,1}^{(i)}(0)=0,\qquad \Delta_{2,1}^{(j)}( \pi/2)=0, \quad i=0,1,\ldots,10, j=0,1,2; \\ &\Delta_{2,2}^{(i)}(0)=0,\qquad \Delta_{2,2}^{(j)}( \pi/2)=0, \quad i=0,1,\ldots,11, j=0,1; \\ &\Delta_{3,1}^{(i)}(0)=0,\qquad \Delta_{3,1}^{(j)}( \pi/2)=0, \quad i=0,1,\ldots,13, j=0,1; \\ &\Delta_{3,2}^{(i)}(0)=0,\qquad \Delta_{3,2}^{(j)}( \pi/2)=0, \quad i=0,1,\ldots,12, j=0,1,2; \end{aligned} $$

we can obtain $P_{1}(x)=x-\frac{1}{6} x^{3}+\frac{1}{120} x^{5}-\frac{1}{5040} x^{7}+\frac{1}{362\mbox{,}880} x^{9} + \frac{\gamma_{1,1}}{{30\mbox{,}240 \pi^{11}}} x^{11} + \frac{\gamma_{1,2}}{{22\mbox{,}680 \pi^{12}}} x^{12}$, $Q_{1}(x)=x-\frac{1}{6} x^{3}+\frac{1}{120} x^{5}-\frac{1}{5040} x^{7}+\frac{1}{362\mbox{,}880} x^{9} - \frac{\gamma_{1,3}}{60\mbox{,}480\pi^{10}} x^{10} +\frac{\gamma_{1,4}}{30\mbox{,}240 \pi^{11}} x^{11} - \frac{\gamma_{1,5}}{45\mbox{,}360 \pi^{12}} x^{12}$, $P_{2}(x)=1-\frac{1}{2} x^{2}+\frac{1}{24} x^{4}-\frac{1}{720} x^{6}+\frac{1}{40\mbox{,}320} x^{8} - \frac{1}{3\mbox{,}628\mbox{,}800} x^{10} + \frac{\gamma_{2,1}}{604\mbox{,}800 \pi^{11}} x^{11} - \frac{\gamma_{2,2}}{302\mbox{,}400 \pi^{12}} x^{12}+ \frac{\gamma_{2,3}}{453\mbox{,}600 \pi^{13}} x^{13}$, $Q_{2}(x)=1-\frac{1}{2} x^{2}+\frac{1}{24} x^{4}-\frac{1}{720} x^{6}+\frac{1}{40\mbox{,}320} x^{8} - \frac{1}{3\mbox{,}628\mbox{,}800} x^{10} + \frac{\gamma_{2,4}}{302\mbox{,}400 \pi^{12}} x^{12}- \frac{\gamma_{2,5}}{226\mbox{,}800\pi^{13}} x^{13}$, $P_{3}(x)=x-\frac{2}{3} x^{3}+\frac{2}{15} x^{5}-\frac{4}{315} x^{7} +\frac{2}{2835} x^{9}-\frac{4}{155\mbox{,}925} x^{11}+ \frac{4}{6\mbox{,}081\mbox{,}075} x^{13}-\frac{\gamma_{3,1}}{6\mbox{,}081\mbox{,}075\pi^{13}} x^{14} + \frac{\gamma_{3,2}}{6\mbox{,}081\mbox{,}075 \pi^{14}} x^{15}$, $Q_{3}(x)=x-\frac{2}{3} x^{3}+\frac{2}{15} x^{5}-\frac{4}{315} x^{7} +\frac{2}{2835} x^{9}-\frac{4}{155\mbox{,}925} x^{11}+ \frac{\gamma_{3,3}}{51\mbox{,}975 \pi^{12}} x^{13}-\frac{\gamma_{3,4}}{ 155\mbox{,}925\pi^{13}} x^{14} + \frac{\gamma_{3,5}}{155\mbox{,}925 \pi^{14}} x^{15}$, where $\gamma_{1,1}= -743\mbox{,}178\mbox{,}240+340\mbox{,}623\mbox{,}360 \pi-11\mbox{,}612\mbox{,}160 \pi^{3}+112\mbox{,}896 \pi^{5}-480 \pi^{7}+\pi^{9}$, $\gamma_{1,2}= -1\mbox{,}021\mbox{,}870\mbox{,}080+464\mbox{,}486\mbox{,}400 \pi-15\mbox{,}482\mbox{,}880 \pi^{3}+145\mbox{,}152 \pi^{5}-576 \pi^{7}+\pi^{9}$, $\gamma_{1,3}= \pi^{9}-960 \pi^{7}+338\mbox{,}688 \pi^{5}-46\mbox{,}448\mbox{,}640 \pi^{3}+7\mbox{,}741\mbox{,}440 \pi^{2}+1\mbox{,}703\mbox{,}116\mbox{,}800 \pi-4\mbox{,}087\mbox{,}480\mbox{,}320$, $\gamma_{1,4}= \pi^{9}-1440 \pi^{7}+564\mbox{,}480 \pi^{5}-81\mbox{,}285\mbox{,}120 \pi^{3}+15\mbox{,}482\mbox{,}880 \pi^{2}+3\mbox{,}065\mbox{,}610\mbox{,}240 \pi-7\mbox{,}431\mbox{,}782\mbox{,}400$, $\gamma_{1,5}= \pi^{9}-1728 \pi^{7}+725\mbox{,}760 \pi^{5}-108\mbox{,}380\mbox{,}160 \pi^{3}+23\mbox{,}224\mbox{,}320 \pi^{2}+4\mbox{,}180\mbox{,}377\mbox{,}600 \pi-10\mbox{,}218\mbox{,}700\mbox{,}800$, $\gamma_{2,1}= \pi^{10}-1200 \pi^{8}+564\mbox{,}480 \pi^{6}-116\mbox{,}121\mbox{,}600 \pi^{4}+8\mbox{,}515\mbox{,}584\mbox{,}000 \pi^{2}+7\mbox{,}431\mbox{,}782\mbox{,}400 \pi-96\mbox{,}613\mbox{,}171\mbox{,}200$, $\gamma_{2,2}= \pi^{10}-1800 \pi^{8}+940\mbox{,}800 \pi^{6}-203\mbox{,}212\mbox{,}800 \pi^{4}+15\mbox{,}328\mbox{,}051\mbox{,}200 \pi^{2}+14\mbox{,}244\mbox{,}249\mbox{,}600 \pi-177\mbox{,}124\mbox{,}147\mbox{,}200$, $\gamma_{2,3}= \pi^{10}-2160 \pi^{8}+ 1\mbox{,}209\mbox{,}600 \pi^{6}-270\mbox{,}950\mbox{,}400 \pi^{4}+20\mbox{,}901\mbox{,}888\mbox{,}000 \pi^{2}+20\mbox{,}437\mbox{,}401\mbox{,}600 \pi-245\mbox{,}248\mbox{,}819\mbox{,}200$, $\gamma_{2,4}= \pi^{1}0-600 \pi^{8}+188\mbox{,}160 \pi^{6}-29\mbox{,}030\mbox{,}400 \pi^{4}+1\mbox{,}703\mbox{,}116\mbox{,}800 \pi^{2}+619\mbox{,}315\mbox{,}200 \pi-16\mbox{,}102\mbox{,}195\mbox{,}200$, $\gamma_{2,5}= \pi^{1}0-720 \pi^{8}+241\mbox{,}920 \pi^{6}-38\mbox{,}707\mbox{,}200 \pi^{4}+2\mbox{,}322\mbox{,}432\mbox{,}000 \pi^{2}+928\mbox{,}972\mbox{,}800 \pi-22\mbox{,}295\mbox{,}347\mbox{,}200$, $\gamma_{3,1}= 16(\pi^{12}-312 \pi^{10}+51\mbox{,}480 \pi^{8}-4\mbox{,}942\mbox{,}080 \pi^{6}+259\mbox{,}459\mbox{,}200 \pi^{4}-6\mbox{,}227\mbox{,}020\mbox{,}800 \pi^{2}+40\mbox{,}475\mbox{,}635\mbox{,}200)$, $\gamma_{3,2}= 16 (\pi^{12}-468 \pi^{10}+85\mbox{,}800 \pi^{8}-8\mbox{,}648\mbox{,}640 \pi^{6}+467\mbox{,}026\mbox{,}560 \pi^{4}-11\mbox{,}416\mbox{,}204\mbox{,}800 \pi^{2}+74\mbox{,}724\mbox{,}249\mbox{,}600)$, $\gamma_{3,3}= 32 (\pi^{10}-275 \pi^{8}+36\mbox{,}960 \pi^{6}-2\mbox{,}494\mbox{,}800 \pi^{4}+73\mbox{,}180\mbox{,}800 \pi^{2}-512\mbox{,}265\mbox{,}600)$, $\gamma_{3,4}= 256(\pi^{10}-330 \pi^{8}+47\mbox{,}520 \pi^{6}-3\mbox{,}326\mbox{,}400 \pi^{4}+99\mbox{,}792\mbox{,}000 \pi^{2}-703\mbox{,}533\mbox{,}600)$, $\gamma_{3,5}= 64(3 \pi^{10}-1100 \pi^{8}+166\mbox{,}320 \pi^{6}-11\mbox{,}975\mbox{,}040 \pi^{4}+365\mbox{,}904\mbox{,}000 \pi^{2}-2\mbox{,}594\mbox{,}592\mbox{,}000)$.

Combining Theorem 1 with Eq. (15), there exists $\eta_{i}(x) \in [0,\pi/2]$, $i=1,2,\ldots,6$, such that

$$\begin{aligned} \Delta_{1,1}(x) &= \frac{\Delta_{1,1}^{(13)}(\eta_{1}(x))}{13!} x^{11}(x- \pi/2)^{2} = \frac{\cos(\eta_{1}(x))}{13!}x^{11} (x-\pi/2)^{2} \geq 0, \quad \forall x \in [0, \pi/2], \\ \Delta_{1,2}(x) &= \frac{\Delta_{1,2}^{(13)}(\eta_{2}(x))}{13!} x^{10}(x- \pi/2)^{3} = \frac{\cos(\eta_{2}(x))}{13!}x^{10} (x-\pi/2)^{3} \leq 0, \quad \forall x \in [0, \pi/2], \\ \Delta_{2,1}(x) &= \frac{\Delta_{2,1}^{(14)}(\eta_{3}(x))}{14!} x^{11} (x- \pi/2)^{3} = \frac{-\cos(\eta_{3}(x))}{14!}x^{11} (x-\pi/2)^{3} \geq 0, \quad \forall x \in [0, \pi/2], \\ \Delta_{2,2}(x) &= \frac{\Delta_{2,2}^{(13)}(\eta_{4}(x))}{13!} x^{11} (x- \pi/2)^{2} = \frac{-\sin(\eta_{4}(x))}{13!}x^{11} (x-\pi/2)^{2} \leq 0, \quad \forall x \in [0, \pi/2], \end{aligned}$$

$$\begin{aligned} \Delta_{3,1}(x) &= \frac{\Delta_{3,1}^{(16)}(2\eta_{5}(x))}{16!} x^{14} (x- \pi/2)^{2} \\ & = \frac{2^{15} \sin(2\eta_{5}(x))}{16!} x^{14}(x-\pi/2)^{2} \geq 0, \quad \forall x \in [0, \pi/2], \\ \Delta_{3,2}(x) &= \frac{\Delta_{3,2}^{(16)}(2\eta_{6}(x))}{16!} x^{13} (x- \pi/2)^{3} \\ & = \frac{2^{15} \sin(2\eta_{6}(x))}{16!} x^{13}(x-\pi/2)^{3} \leq 0, \quad \forall x \in [0, \pi/2]. \end{aligned}$$

So for $\forall x \in [0, \pi/2]$, we have

16 $$ \Delta_{i,1}(x) \geq 0 \quad \mbox{and}\quad \Delta_{i,2}(x) \leq 0, \quad i=1,2,3, $$

i.e., $Q_{1}(x) \geq \sin(x) \geq P_{1}(x)$, $Q_{2}(x) \geq \cos(x) \geq P_{2}(x)$ and $Q_{3}(x) \geq \frac{\sin(2x)}{2} \geq P_{3}(x)$.

(2) Secondly, we prove that $\Delta_{4}(x)=(F(x)-L(x)) \cdot x^{2} \geq 0$, $\forall x \in [0,\pi/2]$, which means that $F(x) \geq L(x)$.

Note that $l_{i}(x)$ and $r_{i}(x)$ are polynomials of degree $3+i$, $i=1,2$, polynomials $P_{1}(x)$, $Q_{1}(x)$, $P_{2}(x)$, $Q_{2}(x)$, $P_{3}(x)$ and $Q_{3}(x)$ are of degree 12, 12, 13, 13, 15 and 15, respectively, by using Maple software, $\forall x \in (0,\pi/2)$, we obtain

17 $$ \begin{aligned} &P_{i}(x) > 0 \quad \mbox{and}\quad Q_{i}(x) > 0, \quad i=1,2,3, \\ &l_{1}(x) \cdot x^{2} > 0 \quad \mbox{and}\quad x-l_{2}(x)\cdot x^{2} > 0, \\ &r_{1}(x) \cdot x^{2} > 0 \quad \mbox{and}\quad x-r_{2}(x) \cdot x^{2} > 0. \end{aligned} $$

Combining Eq. (17) with Eq. (16), we have

18 $$ \begin{aligned}[b] \Delta_{4}(x)&= \sin(x)^{2} \cos(x) - l_{1}(x) x^{2} \cos(x) + \bigl(x- l_{2}(x) x^{2} \bigr) \sin(x) \\ & \geq P_{3}(x) P_{1}(x) - l_{1}(x) x^{2} Q_{2}(x) + \bigl(x- l_{2}(x) x^{2} \bigr) P_{1}(x) \\ & = \frac{(\pi-2 x)^{2} x^{11}}{2\mbox{,}206\mbox{,}700\mbox{,}496\mbox{,}000 (\pi^{4}-180 \pi^{2}+1680)^{2} \pi^{26}} H_{1}(x), \end{aligned} $$

where

$$H_{1}(x)= \sum^{14}_{i=0} \rho_{1,i} x^{i} , $$

and

$$\begin{aligned} &\begin{aligned} \rho_{1,0}&=118\mbox{,}609\mbox{,}920 \bigl(2 \pi^{10}-177 \pi^{8}+4935 \pi^{6}- 85\mbox{,}050 \pi^{4}+831\mbox{,}600 \pi^{2}-3\mbox{,}175\mbox{,}200\bigr) \pi^{23} \\ &>0, \end{aligned} \\ &\begin{aligned} \rho_{1,1}={}&5265 \bigl(40\mbox{,}981 \pi^{19}+8\mbox{,}062\mbox{,}512 \pi^{17}- 1\mbox{,}200 \mbox{,}402\mbox{,}000 \pi^{15}+10\mbox{,}812\mbox{,}049\mbox{,}920 \pi^{13} \\ &{}+1\mbox{,}876\mbox{,}776\mbox{,}249\mbox{,}600 \pi^{11}-245 \mbox{,}548\mbox{,}461\mbox{,}312\mbox{,}000 \pi^{9}+20\mbox{,}600 \mbox{,}900\mbox{,}812\mbox{,}800 \pi^{8} \\ &{}+16\mbox{,}840\mbox{,}163\mbox{,}450\mbox{,}880\mbox{,}000 \pi^{7}-7\mbox{,}416\mbox{,}324\mbox{,}292\mbox{,}608\mbox{,}000 \pi^{6} \\ &{}- 541\mbox{,}159\mbox{,}913\mbox{,}226\mbox{,}240\mbox{,}000 \pi^{5}+736\mbox{,}688\mbox{,}213\mbox{,}065\mbox{,}728\mbox{,}000 \pi^{4} \\ &{} +6\mbox{,}619\mbox{,}069\mbox{,}431\mbox{,}152\mbox{,}640\mbox{,}000 \pi^{3} -12\mbox{,}459\mbox{,}424\mbox{,}811\mbox{,}581\mbox{,}440 \mbox{,}000 \pi^{2} \\ &{}-26\mbox{,}649\mbox{,}325\mbox{,}291\mbox{,}438\mbox{,}080\mbox{,}000 \pi + 58\mbox{,}143\mbox{,}982\mbox{,}454\mbox{,}046\mbox{,}720\mbox{,}000\bigr) \pi^{13}>0, \end{aligned} \\ &\begin{aligned} \rho_{1,2}={}& {-}21\mbox{,}060 \bigl(484 \pi^{21}-1799 \pi^{19}-31\mbox{,}876\mbox{,}698 \pi^{17}+7\mbox{,}133\mbox{,}539\mbox{,}980 \pi^{15} \\ &{}-859\mbox{,}925\mbox{,}324\mbox{,}160 \pi^{13}+60\mbox{,}601 \mbox{,}122\mbox{,}187\mbox{,}200 \pi^{11}-27\mbox{,}467\mbox{,}867 \mbox{,}750\mbox{,}400 \pi^{10} \\ &{}-2\mbox{,}219\mbox{,}580\mbox{,}715\mbox{,}968\mbox{,}000 \pi^{9}+2\mbox{,}419\mbox{,}747\mbox{,}474\mbox{,}636\mbox{,}800 \pi^{8} \\ &{}+41\mbox{,}725\mbox{,}676\mbox{,}095\mbox{,}488\mbox{,}000 \pi^{7}-63\mbox{,}759\mbox{,}788\mbox{,}015\mbox{,}616\mbox{,}000 \pi^{6} \\ &{}-423\mbox{,}071\mbox{,}687\mbox{,}098\mbox{,}368\mbox{,}000 \pi^{5}+769\mbox{,}031\mbox{,}627\mbox{,}341\mbox{,}824\mbox{,}000 \pi^{4} \\ &{}+2\mbox{,}296\mbox{,}485\mbox{,}418\mbox{,}106\mbox{,}880\mbox{,}000 \pi^{3}-4\mbox{,}672\mbox{,}284\mbox{,}304\mbox{,}343\mbox{,}040 \mbox{,}000 \pi^{2} \\ &{}-5\mbox{,}450\mbox{,}998\mbox{,}355\mbox{,}066\mbox{,}880\mbox{,}000 \pi+12 \mbox{,}113\mbox{,}329\mbox{,}677\mbox{,}926\mbox{,}400\mbox{,}000\bigr) \pi^{12}< 0, \end{aligned} \\ &\begin{aligned} \rho_{1,3}={}& 810 \bigl(3287 \pi^{21}-9 \mbox{,}411\mbox{,}072 \pi^{19}+1\mbox{,}953\mbox{,}992\mbox{,}280 \pi^{17}-104\mbox{,}047\mbox{,}433\mbox{,}280 \pi^{15} \\ &{}-4\mbox{,}486\mbox{,}871\mbox{,}592\mbox{,}000 \pi^{13}+792 \mbox{,}548\mbox{,}506\mbox{,}713\mbox{,}600 \pi^{11}-115\mbox{,}307 \mbox{,}819\mbox{,}827\mbox{,}200 \pi^{10} \\ &{}-41\mbox{,}557\mbox{,}678\mbox{,}312\mbox{,}550\mbox{,}400 \pi^{9}+48\mbox{,}741\mbox{,}731\mbox{,}323\mbox{,}084\mbox{,}800 \pi^{8} \\ &{}+730\mbox{,}819\mbox{,}102\mbox{,}261\mbox{,}248\mbox{,}000 \pi^{7}-3\mbox{,}383\mbox{,}354\mbox{,}610\mbox{,}155\mbox{,}520 \mbox{,}000 \pi^{6} \\ &{}-538\mbox{,}971\mbox{,}067\mbox{,}514\mbox{,}880\mbox{,}000 \pi^{5}+61\mbox{,}377\mbox{,}087\mbox{,}827\mbox{,}607\mbox{,}552 \mbox{,}000 \pi^{4} \\ &{}-77\mbox{,}611\mbox{,}833\mbox{,}722\mbox{,}142\mbox{,}720\mbox{,}000 \pi^{3}-404\mbox{,}931\mbox{,}306\mbox{,}376\mbox{,}396\mbox{,}800 \mbox{,}000 \pi^{2} \\ &{}+440\mbox{,}925\mbox{,}200\mbox{,}276\mbox{,}520\mbox{,}960\mbox{,}000 \pi+818\mbox{,}861\mbox{,}086\mbox{,}227\mbox{,}824\mbox{,}640\mbox{,}000\bigr) \pi^{11}< 0, \end{aligned} \\ &\begin{aligned} \rho_{1,4}={}& 42\mbox{,}120\times \bigl(7155 \pi^{21}-3\mbox{,}921\mbox{,}584 \pi^{19}+897\mbox{,}324\mbox{,}984 \pi^{17}-94\mbox{,}307\mbox{,}498\mbox{,}880 \pi^{15} \\ &{}+3\mbox{,}633\mbox{,}527\mbox{,}540\mbox{,}160 \pi^{13}-7 \mbox{,}030\mbox{,}466\mbox{,}150\mbox{,}400 \pi^{12}+28\mbox{,}926 \mbox{,}516\mbox{,}326\mbox{,}400 \pi^{11} \\ &{}+617\mbox{,}046\mbox{,}029\mbox{,}107\mbox{,}200 \pi^{10}-5 \mbox{,}729\mbox{,}151\mbox{,}646\mbox{,}310\mbox{,}400 \pi^{9} \\ &{}-15\mbox{,}296\mbox{,}168\mbox{,}853\mbox{,}504\mbox{,}000 \pi^{8}+163\mbox{,}845\mbox{,}831\mbox{,}131\mbox{,}136\mbox{,}000 \pi^{7} \\ &{}+158\mbox{,}438\mbox{,}094\mbox{,}667\mbox{,}776\mbox{,}000 \pi^{6}-2\mbox{,}245\mbox{,}772\mbox{,}867\mbox{,}272\mbox{,}704 \mbox{,}000 \pi^{5} \\ &{}-381\mbox{,}528\mbox{,}683\mbox{,}053\mbox{,}056\mbox{,}000 \pi^{4}+15\mbox{,}718\mbox{,}487\mbox{,}320\mbox{,}166\mbox{,}400 \mbox{,}000 \pi^{3} \\ &{}-5\mbox{,}595\mbox{,}204\mbox{,}660\mbox{,}756\mbox{,}480\mbox{,}000 \pi^{2} -44\mbox{,}819\mbox{,}319\mbox{,}808\mbox{,}327\mbox{,}680 \mbox{,}000 \pi \\ &{}+33\mbox{,}917\mbox{,}323\mbox{,}098\mbox{,}193\mbox{,}920\mbox{,}000\bigr) \pi^{10}>0, \end{aligned} \\ &\begin{aligned} \rho_{1,5}={}& {-}324 \bigl(3013 \pi^{23}-1 \mbox{,}983\mbox{,}240 \pi^{21}+462\mbox{,}480\mbox{,}560 \pi^{19}-40\mbox{,}847\mbox{,}734\mbox{,}080 \pi^{17} \\ &{}-668\mbox{,}523\mbox{,}878\mbox{,}400 \pi^{15}+303\mbox{,}913 \mbox{,}241\mbox{,}049\mbox{,}600 \pi^{13}-85\mbox{,}019\mbox{,}590 \mbox{,}656\mbox{,}000 \pi^{12} \\ &{}-14\mbox{,}955\mbox{,}232\mbox{,}900\mbox{,}608\mbox{,}000 \pi^{11}+33\mbox{,}853\mbox{,}738\mbox{,}254\mbox{,}336\mbox{,}000 \pi^{10} \\ &{}+452\mbox{,}685\mbox{,}482\mbox{,}016\mbox{,}768\mbox{,}000 \pi^{9}-1\mbox{,}559\mbox{,}110\mbox{,}508\mbox{,}347\mbox{,}392 \mbox{,}000 \pi^{8} \\ &{}-12\mbox{,}135\mbox{,}218\mbox{,}135\mbox{,}040\mbox{,}000\mbox{,}000 \pi^{7}+37\mbox{,}422\mbox{,}223\mbox{,}023\mbox{,}144\mbox{,}960 \mbox{,}000 \pi^{6} \\ &{}+196\mbox{,}234\mbox{,}567\mbox{,}389\mbox{,}020\mbox{,}160\mbox{,}000 \pi^{5}-506\mbox{,}271\mbox{,}257\mbox{,}654\mbox{,}722\mbox{,}560 \mbox{,}000 \pi^{4} \\ &{}-1\mbox{,}522\mbox{,}241\mbox{,}762\mbox{,}859\mbox{,}417\mbox{,}600 \mbox{,}000 \pi^{3}+3\mbox{,}494\mbox{,}407\mbox{,}199\mbox{,}470 \mbox{,}387\mbox{,}200\mbox{,}000 \pi^{2} \\ &{}+4\mbox{,}409\mbox{,}252\mbox{,}002\mbox{,}765\mbox{,}209\mbox{,}600 \mbox{,}000 \pi-9\mbox{,}448\mbox{,}397\mbox{,}148\mbox{,}782\mbox{,}592 \mbox{,}000\mbox{,}000\bigr) \pi^{9}>0, \end{aligned} \\ &\begin{aligned} \rho_{1,6}={}& {-}5616 \bigl(523 \pi^{23}-103 \mbox{,}800 \pi^{21}-73\mbox{,}486\mbox{,}320 \pi^{19}+32 \mbox{,}685\mbox{,}822\mbox{,}720 \pi^{17} \\ &{}-4\mbox{,}913\mbox{,}000\mbox{,}467\mbox{,}200 \pi^{15}-1 \mbox{,}798\mbox{,}491\mbox{,}340\mbox{,}800 \pi^{14}+336\mbox{,}334 \mbox{,}858\mbox{,}675\mbox{,}200 \pi^{13} \\ &{}+147\mbox{,}670\mbox{,}445\mbox{,}260\mbox{,}800 \pi^{12}-11 \mbox{,}847\mbox{,}491\mbox{,}453\mbox{,}952\mbox{,}000 \pi^{11} \\ &{}-167\mbox{,}995\mbox{,}441\mbox{,}152\mbox{,}000 \pi^{10}+267 \mbox{,}060\mbox{,}636\mbox{,}057\mbox{,}600\mbox{,}000 \pi^{9} \\ &{}-118\mbox{,}249\mbox{,}170\mbox{,}665\mbox{,}472\mbox{,}000 \pi^{8}-4\mbox{,}235\mbox{,}673\mbox{,}962\mbox{,}741\mbox{,}760 \mbox{,}000 \pi^{7} \\ &{}+3\mbox{,}896\mbox{,}145\mbox{,}366\mbox{,}220\mbox{,}800\mbox{,}000 \pi^{6}+43\mbox{,}348\mbox{,}415\mbox{,}490\mbox{,}293\mbox{,}760 \mbox{,}000 \pi^{5} \\ &{}-59\mbox{,}726\mbox{,}131\mbox{,}636\mbox{,}469\mbox{,}760\mbox{,}000 \pi^{4}-242\mbox{,}050\mbox{,}284\mbox{,}099\mbox{,}993\mbox{,}600 \mbox{,}000 \pi^{3} \\ &{}+430\mbox{,}888\mbox{,}441\mbox{,}400\mbox{,}524\mbox{,}800\mbox{,}000 \pi^{2}+545\mbox{,}099\mbox{,}835\mbox{,}506\mbox{,}688\mbox{,}000 \mbox{,}000 \pi \\ &{}-1\mbox{,}162\mbox{,}879\mbox{,}649\mbox{,}080\mbox{,}934\mbox{,}400 \mbox{,}000\bigr) \pi^{8}< 0, \end{aligned} \\ &\begin{aligned} \rho_{1,7}={}& 6 \bigl(4603 \pi^{17}-1 \mbox{,}561\mbox{,}248 \pi^{15}+172\mbox{,}972\mbox{,}800 \pi^{13}-1\mbox{,}793\mbox{,}381\mbox{,}990\mbox{,}400 \pi^{9} \\ &{}+144\mbox{,}666\mbox{,}147\mbox{,}225\mbox{,}600 \pi^{7}-114 \mbox{,}776\mbox{,}447\mbox{,}385\mbox{,}600 \pi^{6} \\ &{}-4\mbox{,}787\mbox{,}134\mbox{,}326\mbox{,}374\mbox{,}400 \pi^{5}+5 \mbox{,}624\mbox{,}045\mbox{,}921\mbox{,}894\mbox{,}400 \pi^{4} \\ &{}+74\mbox{,}317\mbox{,}749\mbox{,}682\mbox{,}176\mbox{,}000 \pi^{3}-128\mbox{,}549\mbox{,}621\mbox{,}071\mbox{,}872\mbox{,}000 \pi^{2} \\ &{}-385\mbox{,}648\mbox{,}863\mbox{,}215\mbox{,}616\mbox{,}000 \pi+819 \mbox{,}503\mbox{,}834\mbox{,}333\mbox{,}184\mbox{,}000\bigr) \\ &{}\times \bigl(\pi^{4}-180 \pi^{2}+1680\bigr)^{2} \pi^{7}< 0, \end{aligned} \\ &\begin{aligned} \rho_{1,8}={}& 312 \bigl(199 \pi^{17}-31 \mbox{,}680 \pi^{15}+766\mbox{,}402\mbox{,}560 \pi^{11}-116 \mbox{,}876\mbox{,}390\mbox{,}400 \pi^{9} \\ &{}+7\mbox{,}035\mbox{,}575\mbox{,}500\mbox{,}800 \pi^{7}-4\mbox{,}782 \mbox{,}351\mbox{,}974\mbox{,}400 \pi^{6}-204\mbox{,}560\mbox{,}507 \mbox{,}289\mbox{,}600 \pi^{5} \\ &{}+231\mbox{,}760\mbox{,}134\mbox{,}144\mbox{,}000 \pi^{4}+3 \mbox{,}051\mbox{,}508\mbox{,}432\mbox{,}896\mbox{,}000 \pi^{3} \\ &{}-5\mbox{,}253\mbox{,}229\mbox{,}707\mbox{,}264\mbox{,}000 \pi^{2}-15\mbox{,}759\mbox{,}689\mbox{,}121\mbox{,}792\mbox{,}000 \pi \\ &{}+33\mbox{,}373\mbox{,}459\mbox{,}316\mbox{,}736\mbox{,}000\bigr) \bigl( \pi^{4}-180 \pi^{2}+1680\bigr)^{2} \pi^{6}>0, \end{aligned} \\ &\begin{aligned} \rho_{1,9}={}& {-}12 \bigl(37 \pi^{19}-11 \mbox{,}232 \pi^{17}+484\mbox{,}323\mbox{,}840 \pi^{13}-94 \mbox{,}650\mbox{,}716\mbox{,}160 \pi^{11} \\ &{}+8\mbox{,}146\mbox{,}603\mbox{,}745\mbox{,}280 \pi^{9}-3 \mbox{,}188\mbox{,}234\mbox{,}649\mbox{,}600 \pi^{8}-396\mbox{,}337 \mbox{,}419\mbox{,}878\mbox{,}400 \pi^{7} \\ &{}+267\mbox{,}811\mbox{,}710\mbox{,}566\mbox{,}400 \pi^{6}+11 \mbox{,}398\mbox{,}735\mbox{,}930\mbox{,}982\mbox{,}400 \pi^{5} \\ &{}-12\mbox{,}854\mbox{,}962\mbox{,}107\mbox{,}187\mbox{,}200 \pi^{4}-168\mbox{,}721\mbox{,}377\mbox{,}656\mbox{,}832\mbox{,}000 \pi^{3} \\ &{}+289\mbox{,}236\mbox{,}647\mbox{,}411\mbox{,}712\mbox{,}000 \pi^{2}+867\mbox{,}709\mbox{,}942\mbox{,}235\mbox{,}136\mbox{,}000 \pi \\ &{}-1\mbox{,}831\mbox{,}832\mbox{,}100\mbox{,}274\mbox{,}176\mbox{,}000\bigr) \bigl(\pi^{4}-180 \pi^{2}+1680\bigr)^{2} \pi^{5}>0, \end{aligned} \\ &\begin{aligned} \rho_{1,10}={}& {-}624 \bigl(\pi^{19}-95 \mbox{,}040 \pi^{15}+30\mbox{,}412\mbox{,}800 \pi^{13}-4 \mbox{,}523\mbox{,}904\mbox{,}000 \pi^{11} \\ &{}+353\mbox{,}311\mbox{,}580\mbox{,}160 \pi^{9}-132\mbox{,}843 \mbox{,}110\mbox{,}400 \pi^{8}-16\mbox{,}491\mbox{,}067\mbox{,}084 \mbox{,}800 \pi^{7} \\ &{}+11\mbox{,}036\mbox{,}196\mbox{,}864\mbox{,}000 \pi^{6}+467 \mbox{,}704\mbox{,}826\mbox{,}265\mbox{,}600 \pi^{5}-525\mbox{,}322 \mbox{,}970\mbox{,}726\mbox{,}400 \pi^{4} \\ &{}-6\mbox{,}875\mbox{,}550\mbox{,}646\mbox{,}272\mbox{,}000 \pi^{3}+11\mbox{,}742\mbox{,}513\mbox{,}463\mbox{,}296\mbox{,}000 \pi^{2} \\ &{}+35\mbox{,}227\mbox{,}540\mbox{,}389\mbox{,}888\mbox{,}000 \pi-74\mbox{,}163 \mbox{,}242\mbox{,}926\mbox{,}080\mbox{,}000\bigr) \\ &{}\times \bigl(\pi^{4}-180 \pi^{2}+1680\bigr)^{2} \pi^{4}< 0, \end{aligned} \\ &\begin{aligned} \rho_{1,11}={}& 4 \bigl(\pi^{21}-224 \mbox{,}640 \pi^{17}+87\mbox{,}429\mbox{,}888 \pi^{15}-16 \mbox{,}109\mbox{,}383\mbox{,}680 \pi^{13} \\ &{}+1\mbox{,}712\mbox{,}015\mbox{,}585\mbox{,}280 \pi^{11}-347 \mbox{,}807\mbox{,}416\mbox{,}320 \pi^{10}-119\mbox{,}419\mbox{,}314 \mbox{,}094\mbox{,}080 \pi^{9} \\ &{}+44\mbox{,}635\mbox{,}285\mbox{,}094\mbox{,}400 \pi^{8}+5 \mbox{,}512\mbox{,}856\mbox{,}238\mbox{,}489\mbox{,}600 \pi^{7} \\ &{}-3\mbox{,}672\mbox{,}846\mbox{,}316\mbox{,}339\mbox{,}200 \pi^{6}-155\mbox{,}062\mbox{,}980\mbox{,}417\mbox{,}945\mbox{,}600 \pi^{5} \\ &{}+173\mbox{,}541\mbox{,}988\mbox{,}447\mbox{,}027\mbox{,}200 \pi^{4}+2\mbox{,}265\mbox{,}687\mbox{,}071\mbox{,}391\mbox{,}744 \mbox{,}000 \pi^{3} \\ &{}-3\mbox{,}856\mbox{,}488\mbox{,}632\mbox{,}156\mbox{,}160\mbox{,}000 \pi^{2}-11\mbox{,}569\mbox{,}465\mbox{,}896\mbox{,}468\mbox{,}480 \mbox{,}000 \pi \\ &{}+24\mbox{,}295\mbox{,}878\mbox{,}382\mbox{,}583\mbox{,}808\mbox{,}000\bigr) \bigl(\pi^{4}-180 \pi^{2}+1680\bigr)^{2} \pi^{3}< 0, \end{aligned} \\ &\begin{aligned} \rho_{1,12}={}& 4992 \bigl(\pi^{19}-597 \pi^{17}+175\mbox{,}968 \pi^{15}-29\mbox{,}516\mbox{,}400 \pi^{13}+3\mbox{,}012\mbox{,}992\mbox{,}640 \pi^{11} \\ &{}-603\mbox{,}832\mbox{,}320 \pi^{10}-206\mbox{,}228\mbox{,}151 \mbox{,}360 \pi^{9}+76\mbox{,}640\mbox{,}256\mbox{,}000 \pi^{8} \\ &{}+9\mbox{,}423\mbox{,}877\mbox{,}478\mbox{,}400 \pi^{7}-6\mbox{,}253 \mbox{,}844\mbox{,}889\mbox{,}600 \pi^{6}-263\mbox{,}144\mbox{,}318\mbox{,}976\mbox{,}000 \pi^{5} \\ &{}+293\mbox{,}562\mbox{,}836\mbox{,}582\mbox{,}400 \pi^{4}+3 \mbox{,}824\mbox{,}042\mbox{,}213\mbox{,}376\mbox{,}000 \pi^{3} \\ &{}-6\mbox{,}489\mbox{,}283\mbox{,}756\mbox{,}032\mbox{,}000 \pi^{2}-19\mbox{,}467\mbox{,}851\mbox{,}268\mbox{,}096\mbox{,}000 \pi \\ &{}+40\mbox{,}789\mbox{,}783\mbox{,}609\mbox{,}344\mbox{,}000\bigr) \bigl( \pi^{4}-180 \pi^{2}+1680\bigr)^{2} \pi^{2}>0, \end{aligned} \\ &\begin{aligned} \rho_{1,13}={}& {-}48 \bigl(\pi^{21}-740 \pi^{19}+257\mbox{,}768 \pi^{17}-52\mbox{,}788\mbox{,}672 \pi^{15}+7\mbox{,}093\mbox{,}975\mbox{,}680 \pi^{13} \\ &{}-743\mbox{,}178\mbox{,}240 \pi^{12}-678\mbox{,}927\mbox{,}674 \mbox{,}880 \pi^{11}+135\mbox{,}258\mbox{,}439\mbox{,}680 \pi^{10} \\ &{}+45\mbox{,}959\mbox{,}564\mbox{,}851\mbox{,}200 \pi^{9}-17 \mbox{,}003\mbox{,}918\mbox{,}131\mbox{,}200 \pi^{8}-2\mbox{,}082 \mbox{,}714\mbox{,}284\mbox{,}851\mbox{,}200 \pi^{7} \\ &{}+1\mbox{,}377\mbox{,}317\mbox{,}368\mbox{,}627\mbox{,}200 \pi^{6}+57\mbox{,}780\mbox{,}376\mbox{,}554\mbox{,}700\mbox{,}800 \pi^{5} \\ &{}-64\mbox{,}274\mbox{,}810\mbox{,}535\mbox{,}936\mbox{,}000 \pi^{4}-835\mbox{,}572\mbox{,}536\mbox{,}967\mbox{,}168\mbox{,}000 \pi^{3} \\ &{}+1\mbox{,}414\mbox{,}045\mbox{,}831\mbox{,}790\mbox{,}592\mbox{,}000 \pi^{2}+4\mbox{,}242\mbox{,}137\mbox{,}495\mbox{,}371\mbox{,}776 \mbox{,}000 \pi \\ &{}-8\mbox{,}869\mbox{,}923\mbox{,}853\mbox{,}959\mbox{,}168\mbox{,}000\bigr) \bigl(\pi^{4}-180 \pi^{2}+1680\bigr)^{2} \pi>0, \end{aligned} \\ &\begin{aligned} \rho_{1,14}={}& 64 \bigl(\pi^{9}-576 \pi^{7}+145\mbox{,}152 \pi^{5}-15\mbox{,}482\mbox{,}880 \pi^{3}+464\mbox{,}486\mbox{,}400 \pi \\ &{}-1\mbox{,}021\mbox{,}870\mbox{,}080\bigr) \bigl(\pi^{12}-468 \pi^{10}+85\mbox{,}800 \pi^{8}-8\mbox{,}648\mbox{,}640 \pi^{6}+467\mbox{,}026\mbox{,}560 \pi^{4} \\ &{}-11\mbox{,}416\mbox{,}204\mbox{,}800 \pi^{2}+74\mbox{,}724 \mbox{,}249\mbox{,}600\bigr) \bigl(\pi^{4}-180 \pi^{2}+1680 \bigr)^{2}< 0. \end{aligned} \end{aligned}$$

Note that $0< x^{i}<(\frac{\pi}{2})^{i}, i=2,3, \forall x \in (0,\pi/2)$, we have $H_{1}(x) \geq (\rho_{1,0} + \rho_{1,2} \cdot (\frac{\pi}{2})^{2}+\rho_{1,3}\cdot (\frac{\pi}{2})^{3}) + \rho_{1,1} x + (\rho_{1,4}+\rho_{1,6}\cdot (\frac{\pi}{2})^{2}+\rho_{1,7}\cdot (\frac{\pi}{2})^{3}) x^{4} + \rho_{1,5} x^{5} + (\rho_{1,8}+\rho_{1,10} \cdot (\frac{\pi }{2})^{2}+\rho_{1,11} \cdot (\frac{\pi}{2})^{3}) x^{8} + \rho_{1,9} x^{9} + (\rho_{1,12}+\rho_{1,14}\cdot (\frac{\pi}{2})^{2}) x^{12} + \rho_{1,13} x^{13} \approx 9.6 \cdot 10^{8} x^{13}+4.3 \cdot 10^{9}*x^{1}2+1.5 \cdot 10^{13} x^{9}+5.0 \cdot 10^{13} x^{8}+4.2 \cdot 10^{16} x^{5}+1.2 \cdot 10^{17} x^{4}+1.5 \cdot 10^{19} x+3.8 \cdot 10^{19} > 0$, $\forall x \in (0, \pi/2)$. It leads to $\Delta_{4}(x) \geq 0$ and $F(x) \geq L(x)$, $\forall x \in [0, \pi/2]$.

(3) Finally, we prove that $\Delta_{5}(x)=(F(x)-R(x)) \cdot x^{2} \leq 0$, $\forall x \in [0,\pi/2]$, which means that $F(x) \leq R(x)$. Combining Eq. (17) with Eq. (16), we have

19 $$ \begin{aligned}[b] \Delta_{5}(x)&= \sin(x)^{2} \cos(x) - r_{1}(x) x^{2} \cos(x) + \bigl(x- r_{2}(x) x^{2} \bigr) \sin(x) \\ & \leq Q_{3}(x) Q_{1}(x) - r_{1}(x) x^{2} P_{2}(x) + \bigl(x- r_{2}(x) x^{2} \bigr) Q_{1}(x) \\ & \triangleq \frac{ (\pi-2 x)^{3} x^{10}}{-56\mbox{,}582\mbox{,}064\mbox{,}000 \bar{\gamma} \pi^{26}} H_{2}(x), \end{aligned} $$

where

$$\begin{aligned} &\bar{\gamma} = 5 \pi^{10}-558 \pi^{8}+12\mbox{,}480 \pi^{6}-177\mbox{,}120 \pi^{4}+1\mbox{,}756\mbox{,}800 \pi^{2}-7\mbox{,}257\mbox{,}600 \approx -0.12 < 0, \\ &H_{2}(x)= \sum^{14}_{i=0} \rho_{2,i} x^{i}, \end{aligned}$$

and

$$\begin{aligned} &\begin{aligned} \rho_{2,0}={}& {-}54\mbox{,}743\mbox{,}040 \bigl(10 \pi^{14}-1542 \pi^{12}+72\mbox{,}615 \pi^{10}-1 \mbox{,}559\mbox{,}565 \pi^{8}+14\mbox{,}049\mbox{,}000 \pi^{6} \\ &{}-5\mbox{,}896\mbox{,}800 \pi^{4}-635\mbox{,}040\mbox{,}000 \pi^{2}+2\mbox{,}667\mbox{,}168\mbox{,}000\bigr) \pi^{19}< 0, \end{aligned} \\ &\begin{aligned} \rho_{2,1}={}& {-}17\mbox{,}820 \bigl(187\mbox{,}379 \pi^{19}-27\mbox{,}905\mbox{,}634 \pi^{17}+1\mbox{,}101 \mbox{,}819\mbox{,}840 \pi^{15} \\ &{}+16\mbox{,}808\mbox{,}329\mbox{,}440 \pi^{13}-4\mbox{,}064 \mbox{,}256\mbox{,}000 \pi^{12}-3\mbox{,}819\mbox{,}046\mbox{,}492 \mbox{,}800 \pi^{11} \\ &{}+2\mbox{,}599\mbox{,}498\mbox{,}137\mbox{,}600 \pi^{10}+167 \mbox{,}022\mbox{,}175\mbox{,}219\mbox{,}200 \pi^{9} \\ &{}-249\mbox{,}629\mbox{,}854\mbox{,}924\mbox{,}800 \pi^{8} \\ &{}-3\mbox{,}170\mbox{,}509\mbox{,}848\mbox{,}576\mbox{,}000 \pi^{7}+5 \mbox{,}500\mbox{,}206\mbox{,}415\mbox{,}872\mbox{,}000 \pi^{6} \\ &{}+40\mbox{,}549\mbox{,}244\mbox{,}682\mbox{,}240\mbox{,}000 \pi^{5}-77\mbox{,}445\mbox{,}340\mbox{,}987\mbox{,}392\mbox{,}000 \pi^{4} \\ &{}-349\mbox{,}559\mbox{,}830\mbox{,}609\mbox{,}920\mbox{,}000 \pi^{3}+759\mbox{,}892\mbox{,}318\mbox{,}617\mbox{,}600\mbox{,}000 \pi^{2} \\ &{}+1\mbox{,}297\mbox{,}856\mbox{,}751\mbox{,}206\mbox{,}400\mbox{,}000 \pi-3 \mbox{,}114\mbox{,}856\mbox{,}202\mbox{,}895\mbox{,}360\mbox{,}000\bigr) \pi^{13}< 0, \end{aligned} \\ &\begin{aligned} \rho_{2,2}={}& 135 \bigl(470\mbox{,}935 \pi^{21}-163\mbox{,}016\mbox{,}586 \pi^{19}+16\mbox{,}583 \mbox{,}895\mbox{,}072 \pi^{17} \\ &{}-399\mbox{,}774\mbox{,}876\mbox{,}480 \pi^{15}-39\mbox{,}075 \mbox{,}261\mbox{,}120\mbox{,}000 \pi^{13} \\ &{}+7\mbox{,}081\mbox{,}559\mbox{,}654\mbox{,}400 \pi^{12}+2 \mbox{,}891\mbox{,}256\mbox{,}628\mbox{,}377\mbox{,}600 \pi^{11} \\ &{}-4\mbox{,}039\mbox{,}064\mbox{,}115\mbox{,}609\mbox{,}600 \pi^{10}-54\mbox{,}681\mbox{,}296\mbox{,}556\mbox{,}902\mbox{,}400 \pi^{9} \\ &{}+116\mbox{,}088\mbox{,}651\mbox{,}192\mbox{,}729\mbox{,}600 \pi^{8}+29\mbox{,}143\mbox{,}836\mbox{,}868\mbox{,}608\mbox{,}000 \pi^{7} \\ &{}-519\mbox{,}915\mbox{,}234\mbox{,}263\mbox{,}040\mbox{,}000 \pi^{6}+10\mbox{,}877\mbox{,}661\mbox{,}896\mbox{,}048\mbox{,}640 \mbox{,}000 \pi^{5} \\ &{}-19\mbox{,}708\mbox{,}263\mbox{,}780\mbox{,}581\mbox{,}376\mbox{,}000 \pi^{4}-126\mbox{,}300\mbox{,}002\mbox{,}703\mbox{,}114\mbox{,}240 \mbox{,}000 \pi^{3} \\ &{}+281\mbox{,}041\mbox{,}609\mbox{,}068\mbox{,}380\mbox{,}160\mbox{,}000 \pi^{2}+445\mbox{,}424\mbox{,}437\mbox{,}014\mbox{,}036\mbox{,}480 \mbox{,}000 \pi \\ &{}-1\mbox{,}083\mbox{,}969\mbox{,}958\mbox{,}607\mbox{,}585\mbox{,}280 \mbox{,}000\bigr) \pi^{12}>0, \end{aligned} \\ &\begin{aligned} \rho_{2,3}={}& 3240 \bigl(118\mbox{,}457 \pi^{21}-29\mbox{,}814\mbox{,}542 \pi^{19}+2\mbox{,}683 \mbox{,}328\mbox{,}595 \pi^{17}-112\mbox{,}738\mbox{,}193\mbox{,}340 \pi^{15} \\ &{}-3\mbox{,}193\mbox{,}344\mbox{,}000 \pi^{14}+3\mbox{,}713 \mbox{,}195\mbox{,}298\mbox{,}960 \pi^{13}+4\mbox{,}257\mbox{,}366 \mbox{,}220\mbox{,}800 \pi^{12} \\ &{}-176\mbox{,}445\mbox{,}931\mbox{,}032\mbox{,}000 \pi^{11}-538 \mbox{,}724\mbox{,}796\mbox{,}825\mbox{,}600 \pi^{10} \\ &{}+5\mbox{,}029\mbox{,}126\mbox{,}333\mbox{,}862\mbox{,}400 \pi^{9}+12\mbox{,}839\mbox{,}971\mbox{,}273\mbox{,}113\mbox{,}600 \pi^{8} \\ &{}-58\mbox{,}269\mbox{,}701\mbox{,}597\mbox{,}184\mbox{,}000 \pi^{7}-255\mbox{,}180\mbox{,}783\mbox{,}255\mbox{,}552\mbox{,}000 \pi^{6} \\ &{}+377\mbox{,}530\mbox{,}820\mbox{,}739\mbox{,}072\mbox{,}000 \pi^{5}+3\mbox{,}806\mbox{,}634\mbox{,}452\mbox{,}189\mbox{,}184 \mbox{,}000 \pi^{4} \\ &{}-2\mbox{,}922\mbox{,}752\mbox{,}802\mbox{,}816\mbox{,}000\mbox{,}000 \pi^{3}-26\mbox{,}758\mbox{,}510\mbox{,}065\mbox{,}745\mbox{,}920 \mbox{,}000 \pi^{2} \\ &{}+14\mbox{,}276\mbox{,}424\mbox{,}263\mbox{,}270\mbox{,}400\mbox{,}000 \pi+63 \mbox{,}335\mbox{,}409\mbox{,}458\mbox{,}872\mbox{,}320\mbox{,}000\bigr) \pi^{11}>0, \end{aligned} \\ &\begin{aligned} \rho_{2,4}={}& {-}1350 \bigl(2579 \pi^{23}-1 \mbox{,}304\mbox{,}586 \pi^{21}+190\mbox{,}808\mbox{,}712 \pi^{19}-4\mbox{,}857\mbox{,}853\mbox{,}680 \pi^{17} \\ &{}-823\mbox{,}686\mbox{,}670\mbox{,}080 \pi^{15}+272\mbox{,}839 \mbox{,}311\mbox{,}360 \pi^{14}+28\mbox{,}966\mbox{,}274\mbox{,}628 \mbox{,}480 \pi^{13} \\ &{}-219\mbox{,}724\mbox{,}548\mbox{,}341\mbox{,}760 \pi^{12}+166 \mbox{,}206\mbox{,}481\mbox{,}943\mbox{,}040 \pi^{11} \\ &{}+5\mbox{,}303\mbox{,}591\mbox{,}552\mbox{,}286\mbox{,}720 \pi^{10}-1\mbox{,}171\mbox{,}730\mbox{,}648\mbox{,}309\mbox{,}760 \pi^{9} \\ &{}+2\mbox{,}317\mbox{,}601\mbox{,}341\mbox{,}440\mbox{,}000 \pi^{8}-576\mbox{,}193\mbox{,}032\mbox{,}614\mbox{,}707\mbox{,}200 \pi^{7} \\ &{}-1\mbox{,}263\mbox{,}206\mbox{,}036\mbox{,}039\mbox{,}270\mbox{,}400 \pi^{6}+15\mbox{,}415\mbox{,}077\mbox{,}252\mbox{,}995\mbox{,}481 \mbox{,}600 \pi^{5} \\ &{}+10\mbox{,}344\mbox{,}536\mbox{,}334\mbox{,}139\mbox{,}392\mbox{,}000 \pi^{4}-149\mbox{,}767\mbox{,}724\mbox{,}873\mbox{,}023\mbox{,}488 \mbox{,}000 \pi^{3} \\ &{}+25\mbox{,}334\mbox{,}163\mbox{,}783\mbox{,}548\mbox{,}928\mbox{,}000 \pi^{2}+507\mbox{,}098\mbox{,}589\mbox{,}831\mbox{,}364\mbox{,}608 \mbox{,}000 \pi \\ &{}-361\mbox{,}323\mbox{,}319\mbox{,}535\mbox{,}861\mbox{,}760\mbox{,}000\bigr) \pi^{10}< 0, \end{aligned} \\ &\begin{aligned} \rho_{2,5}={}& {-}43\mbox{,}200 \bigl(498 \pi^{23}-177\mbox{,}844 \pi^{21}+26\mbox{,}426\mbox{,}133 \pi^{19}-1\mbox{,}772\mbox{,}952\mbox{,}897 \pi^{17} \\ &{}+143\mbox{,}700\mbox{,}480 \pi^{16}+13\mbox{,}733\mbox{,}029 \mbox{,}656 \pi^{15}-119\mbox{,}156\mbox{,}438\mbox{,}016 \pi^{14} \\ &{}+4\mbox{,}355\mbox{,}924\mbox{,}207\mbox{,}040 \pi^{13}+7 \mbox{,}627\mbox{,}468\mbox{,}197\mbox{,}888 \pi^{12}-240\mbox{,}282 \mbox{,}095\mbox{,}518\mbox{,}080 \pi^{11} \\ &{}-77\mbox{,}912\mbox{,}484\mbox{,}249\mbox{,}600 \pi^{10}+5 \mbox{,}350\mbox{,}872\mbox{,}626\mbox{,}668\mbox{,}800 \pi^{9} \\ &{}-3\mbox{,}604\mbox{,}409\mbox{,}633\mbox{,}341\mbox{,}440 \pi^{8}-61\mbox{,}395\mbox{,}244\mbox{,}517\mbox{,}376\mbox{,}000 \pi^{7} \\ &{}+111\mbox{,}158\mbox{,}598\mbox{,}116\mbox{,}966\mbox{,}400 \pi^{6}+395\mbox{,}514\mbox{,}763\mbox{,}370\mbox{,}496\mbox{,}000 \pi^{5} \\ &{}-1\mbox{,}303\mbox{,}336\mbox{,}590\mbox{,}822\mbox{,}604\mbox{,}800 \pi^{4}-1\mbox{,}470\mbox{,}286\mbox{,}291\mbox{,}009\mbox{,}536 \mbox{,}000 \pi^{3} \\ &{}+7\mbox{,}275\mbox{,}723\mbox{,}144\mbox{,}560\mbox{,}640\mbox{,}000 \pi^{2}+2\mbox{,}725\mbox{,}499\mbox{,}177\mbox{,}533\mbox{,}440 \mbox{,}000 \pi \\ &{}-16\mbox{,}197\mbox{,}252\mbox{,}255\mbox{,}055\mbox{,}872\mbox{,}000\bigr) \pi^{9}< 0, \end{aligned} \\ &\begin{aligned} \rho_{2,6}={}& 36 \bigl(3055 \pi^{25}-1 \mbox{,}943\mbox{,}682 \pi^{23}+419\mbox{,}154\mbox{,}420 \pi^{21}-44\mbox{,}118\mbox{,}680\mbox{,}220 \pi^{19} \\ &{}+2\mbox{,}140\mbox{,}947\mbox{,}712\mbox{,}800 \pi^{17}-130 \mbox{,}288\mbox{,}435\mbox{,}200 \pi^{16}+86\mbox{,}530\mbox{,}606 \mbox{,}128\mbox{,}000 \pi^{15} \\ &{}+3\mbox{,}142\mbox{,}250\mbox{,}496\mbox{,}000 \pi^{14}-20 \mbox{,}960\mbox{,}647\mbox{,}460\mbox{,}121\mbox{,}600 \pi^{13} \\ &{}-17\mbox{,}423\mbox{,}671\mbox{,}703\mbox{,}961\mbox{,}600 \pi^{12}+1\mbox{,}023\mbox{,}195\mbox{,}300\mbox{,}994\mbox{,}944 \mbox{,}000 \pi^{11} \\ &{}+344\mbox{,}467\mbox{,}294\mbox{,}617\mbox{,}600\mbox{,}000 \pi^{10}-23\mbox{,}018\mbox{,}674\mbox{,}839\mbox{,}164\mbox{,}928 \mbox{,}000 \pi^{9} \\ &{}+5\mbox{,}473\mbox{,}622\mbox{,}558\mbox{,}638\mbox{,}080\mbox{,}000 \pi^{8}+281\mbox{,}073\mbox{,}089\mbox{,}819\mbox{,}934\mbox{,}720 \mbox{,}000 \pi^{7} \\ &{}-255\mbox{,}696\mbox{,}320\mbox{,}798\mbox{,}392\mbox{,}320\mbox{,}000 \pi^{6}-1\mbox{,}893\mbox{,}832\mbox{,}571\mbox{,}360\mbox{,}378 \mbox{,}880\mbox{,}000 \pi^{5} \\ &{}+3\mbox{,}403\mbox{,}944\mbox{,}523\mbox{,}821\mbox{,}219\mbox{,}840 \mbox{,}000 \pi^{4}+6\mbox{,}320\mbox{,}191\mbox{,}562\mbox{,}160 \mbox{,}537\mbox{,}600\mbox{,}000 \pi^{3} \\ &{}-20\mbox{,}096\mbox{,}013\mbox{,}935\mbox{,}679\mbox{,}897\mbox{,}600 \mbox{,}000 \pi^{2}-7\mbox{,}101\mbox{,}872\mbox{,}142\mbox{,}601 \mbox{,}420\mbox{,}800\mbox{,}000 \pi \\ &{}+44\mbox{,}480\mbox{,}146\mbox{,}577\mbox{,}345\mbox{,}740\mbox{,}800 \mbox{,}000\bigr) \pi^{8}>0, \end{aligned} \\ &\begin{aligned} \rho_{2,7}={}& 864 \bigl(5 \pi^{10}-558 \pi^{8}+12\mbox{,}480 \pi^{6}-177\mbox{,}120 \pi^{4}+1\mbox{,}756\mbox{,}800 \pi^{2} \\ &{}-7\mbox{,}257\mbox{,}600\bigr) \bigl(139 \pi^{15}-30\mbox{,}800 \pi^{13}+638\mbox{,}668\mbox{,}800 \pi^{9}-106\mbox{,}444 \mbox{,}800 \pi^{8} \\ &{}-64\mbox{,}399\mbox{,}104\mbox{,}000 \pi^{7}+60\mbox{,}673 \mbox{,}536\mbox{,}000 \pi^{6}+2\mbox{,}557\mbox{,}229\mbox{,}875 \mbox{,}200 \pi^{5} \\ &{}-3\mbox{,}344\mbox{,}069\mbox{,}836\mbox{,}800 \pi^{4}-45 \mbox{,}600\mbox{,}952\mbox{,}320\mbox{,}000 \pi^{3}+86\mbox{,}373 \mbox{,}568\mbox{,}512\mbox{,}000 \pi^{2} \\ &{}+255\mbox{,}365\mbox{,}332\mbox{,}992\mbox{,}000 \pi-579\mbox{,}400 \mbox{,}335\mbox{,}360\mbox{,}000\bigr) \pi^{7}>0, \end{aligned} \\ &\begin{aligned} \rho_{2,8}={}& {-}2 \bigl(199 \pi^{17}-95 \mbox{,}040 \pi^{15}+5\mbox{,}364\mbox{,}817\mbox{,}920 \pi^{11}-1\mbox{,}051\mbox{,}887\mbox{,}513\mbox{,}600 \pi^{9} \\ &{}+91\mbox{,}968\mbox{,}307\mbox{,}200 \pi^{8}+77\mbox{,}391 \mbox{,}330\mbox{,}508\mbox{,}800 \pi^{7}-61\mbox{,}250\mbox{,}892 \mbox{,}595\mbox{,}200 \pi^{6} \\ &{}-2\mbox{,}652\mbox{,}044\mbox{,}090\mbox{,}572\mbox{,}800 \pi^{5}+3 \mbox{,}321\mbox{,}895\mbox{,}256\mbox{,}064\mbox{,}000 \pi^{4} \\ &{}+45\mbox{,}115\mbox{,}972\mbox{,}780\mbox{,}032\mbox{,}000 \pi^{3}-84\mbox{,}515\mbox{,}195\mbox{,}584\mbox{,}512\mbox{,}000 \pi^{2} \\ &{}-250\mbox{,}300\mbox{,}944\mbox{,}875\mbox{,}520\mbox{,}000 \pi+563 \mbox{,}640\mbox{,}646\mbox{,}238\mbox{,}208\mbox{,}000\bigr) \bigl(5 \pi^{10}-558 \pi^{8} \\ &{}+12\mbox{,}480 \pi^{6}-177\mbox{,}120 \pi^{4}+1 \mbox{,}756\mbox{,}800 \pi^{2}-7\mbox{,}257\mbox{,}600\bigr) \pi^{6}< 0, \end{aligned} \\ &\begin{aligned} \rho_{2,9}={}& {-}1728 \bigl(5 \pi^{10}-558 \pi^{8}+12\mbox{,}480 \pi^{6}-177\mbox{,}120 \pi^{4}+1\mbox{,}756\mbox{,}800 \pi^{2}-7\mbox{,}257 \mbox{,}600\bigr) \\ &{}\times\bigl(\pi^{17}-129\mbox{,}360 \pi^{13}+33 \mbox{,}707\mbox{,}520 \pi^{11}-2\mbox{,}956\mbox{,}800 \pi^{10}-3\mbox{,}626\mbox{,}515\mbox{,}200 \pi^{9} \\ &{}+1\mbox{,}774\mbox{,}080\mbox{,}000 \pi^{8}+211\mbox{,}718 \mbox{,}707\mbox{,}200 \pi^{7}-163\mbox{,}924\mbox{,}992\mbox{,}000 \pi^{6} \\ &{}-7\mbox{,}086\mbox{,}030\mbox{,}336\mbox{,}000 \pi^{5}+8\mbox{,}762 \mbox{,}535\mbox{,}936\mbox{,}000 \pi^{4}+118\mbox{,}562\mbox{,}476 \mbox{,}032\mbox{,}000 \pi^{3} \\ &{}-219\mbox{,}957\mbox{,}534\mbox{,}720\mbox{,}000 \pi^{2}-652 \mbox{,}361\mbox{,}859\mbox{,}072\mbox{,}000 \pi \\ &{}+1\mbox{,}459\mbox{,}230\mbox{,}474\mbox{,}240\mbox{,}000\bigr) \pi^{5}< 0, \end{aligned} \\ &\begin{aligned} \rho_{2,10}={}& 4 \bigl(5 \pi^{10}-558 \pi^{8}+12\mbox{,}480 \pi^{6}-177\mbox{,}120 \pi^{4}+1\mbox{,}756\mbox{,}800 \pi^{2}-7\mbox{,}257 \mbox{,}600\bigr) \\ &{}\times \bigl(\pi^{19}-475\mbox{,}200 \pi^{15}+212 \mbox{,}889\mbox{,}600 \pi^{13}-40\mbox{,}715\mbox{,}136\mbox{,}000 \pi^{11} \\ &{}+2\mbox{,}554\mbox{,}675\mbox{,}200 \pi^{10}+3\mbox{,}886 \mbox{,}427\mbox{,}381\mbox{,}760 \pi^{9}-1\mbox{,}778\mbox{,}053 \mbox{,}939\mbox{,}200 \pi^{8} \\ &{}-214\mbox{,}297\mbox{,}651\mbox{,}814\mbox{,}400 \pi^{7}+162 \mbox{,}232\mbox{,}093\mbox{,}900\mbox{,}800 \pi^{6} \\ &{}+6\mbox{,}999\mbox{,}156\mbox{,}051\mbox{,}148\mbox{,}800 \pi^{5}-8 \mbox{,}559\mbox{,}674\mbox{,}287\mbox{,}718\mbox{,}400 \pi^{4} \\ &{}-115\mbox{,}416\mbox{,}546\mbox{,}803\mbox{,}712\mbox{,}000 \pi^{3}+212\mbox{,}292\mbox{,}282\mbox{,}875\mbox{,}904\mbox{,}000 \pi^{2} \\ &{}+630\mbox{,}387\mbox{,}564\mbox{,}871\mbox{,}680\mbox{,}000 \pi-1\mbox{,}401 \mbox{,}685\mbox{,}291\mbox{,}302\mbox{,}912\mbox{,}000\bigr) \pi^{4}>0, \end{aligned} \\ &\begin{aligned} \rho_{2,11}={}& 4608 \bigl(5 \pi^{10}-558 \pi^{8}+12\mbox{,}480 \pi^{6}-177\mbox{,}120 \pi^{4}+1\mbox{,}756\mbox{,}800 \pi^{2}-7\mbox{,}257 \mbox{,}600\bigr) \\ &{}\times \bigl(5 \pi^{17}-2919 \pi^{15}+717\mbox{,}120 \pi^{13}-40\mbox{,}320 \pi^{12}-95\mbox{,}264\mbox{,}400 \pi^{11} \\ &{}+25\mbox{,}724\mbox{,}160 \pi^{10}+7\mbox{,}974\mbox{,}046 \mbox{,}080 \pi^{9}-3\mbox{,}548\mbox{,}160\mbox{,}000 \pi^{8}-429\mbox{,}305\mbox{,}184\mbox{,}000 \pi^{7} \\ &{}+319\mbox{,}973\mbox{,}068\mbox{,}800 \pi^{6}+13\mbox{,}775 \mbox{,}287\mbox{,}680\mbox{,}000 \pi^{5}-16\mbox{,}684\mbox{,}583 \mbox{,}731\mbox{,}200 \pi^{4} \\ &{}-224\mbox{,}249\mbox{,}389\mbox{,}056\mbox{,}000 \pi^{3}+409 \mbox{,}335\mbox{,}607\mbox{,}296\mbox{,}000 \pi^{2} \\ &{}+1\mbox{,}216\mbox{,}740\mbox{,}704\mbox{,}256\mbox{,}000 \pi-2\mbox{,}690 \mbox{,}992\mbox{,}668\mbox{,}672\mbox{,}000\bigr) \pi^{3}>0, \end{aligned} \\ &\begin{aligned} \rho_{2,12}= {}&{-}96 \bigl(5 \pi^{10}-558 \pi^{8}+12\mbox{,}480 \pi^{6}-177\mbox{,}120 \pi^{4}+1\mbox{,}756\mbox{,}800 \pi^{2}-7\mbox{,}257 \mbox{,}600\bigr) \\ &{}\times \bigl(\pi^{19}-995 \pi^{17}+410\mbox{,}592 \pi^{15}-88\mbox{,}549\mbox{,}200 \pi^{13}+3\mbox{,}870 \mbox{,}720 \pi^{12} \\ &{}+11\mbox{,}047\mbox{,}639\mbox{,}680 \pi^{11}-2\mbox{,}841 \mbox{,}108\mbox{,}480 \pi^{10}-893\mbox{,}605\mbox{,}426\mbox{,}560 \pi^{9} \\ &{}+388\mbox{,}310\mbox{,}630\mbox{,}400 \pi^{8}+47\mbox{,}103 \mbox{,}101\mbox{,}337\mbox{,}600 \pi^{7}-34\mbox{,}641\mbox{,}395 \mbox{,}712\mbox{,}000 \pi^{6} \\ &{}-1\mbox{,}488\mbox{,}093\mbox{,}194\mbox{,}649\mbox{,}600 \pi^{5}+1 \mbox{,}787\mbox{,}128\mbox{,}145\mbox{,}510\mbox{,}400 \pi^{4} \\ &{}+23\mbox{,}948\mbox{,}547\mbox{,}194\mbox{,}880\mbox{,}000 \pi^{3}-43\mbox{,}416\mbox{,}398\mbox{,}462\mbox{,}976\mbox{,}000 \pi^{2} \\ &{}-129\mbox{,}167\mbox{,}648\mbox{,}096\mbox{,}256\mbox{,}000 \pi+284 \mbox{,}292\mbox{,}431\mbox{,}216\mbox{,}640\mbox{,}000\bigr) \pi^{2}< 0, \end{aligned} \\ &\begin{aligned} \rho_{2,13}= {}&256 \bigl(5 \pi^{10}-558 \pi^{8}+12\mbox{,}480 \pi^{6}-177\mbox{,}120 \pi^{4}+1\mbox{,}756\mbox{,}800 \pi^{2}-7\mbox{,}257 \mbox{,}600\bigr) \\ &{}\times \bigl(\pi^{19}-1734 \pi^{17}+1\mbox{,}043 \mbox{,}280 \pi^{15}-318\mbox{,}349\mbox{,}440 \pi^{13}+14 \mbox{,}515\mbox{,}200 \pi^{12} \\ &{}+56\mbox{,}498\mbox{,}601\mbox{,}600 \pi^{11}-11\mbox{,}554 \mbox{,}099\mbox{,}200 \pi^{10}-6\mbox{,}203\mbox{,}534\mbox{,}752 \mbox{,}800 \pi^{9} \\ &{}+2\mbox{,}843\mbox{,}353\mbox{,}497\mbox{,}600 \pi^{8}+419 \mbox{,}195\mbox{,}855\mbox{,}232\mbox{,}000 \pi^{7}-354\mbox{,}261 \mbox{,}919\mbox{,}334\mbox{,}400 \pi^{6} \\ &{}-16\mbox{,}350\mbox{,}918\mbox{,}880\mbox{,}665\mbox{,}600 \pi^{5}+22\mbox{,}731\mbox{,}806\mbox{,}490\mbox{,}624\mbox{,}000 \pi^{4} \\ &{}+314\mbox{,}092\mbox{,}921\mbox{,}798\mbox{,}656\mbox{,}000 \pi^{3}-646\mbox{,}147\mbox{,}253\mbox{,}993\mbox{,}472\mbox{,}000 \pi^{2} \\ &{}-1\mbox{,}856\mbox{,}398\mbox{,}674\mbox{,}493\mbox{,}440\mbox{,}000 \pi+4 \mbox{,}477\mbox{,}605\mbox{,}791\mbox{,}662\mbox{,}080\mbox{,}000\bigr) \pi< 0, \end{aligned} \\ &\begin{aligned} \rho_{2,14}= {}&{-}64 \bigl(\pi^{9}-1728 \pi^{7}+725\mbox{,}760 \pi^{5}-108\mbox{,}380\mbox{,}160 \pi^{3}+23\mbox{,}224\mbox{,}320 \pi^{2} \\ &{}+4\mbox{,}180\mbox{,}377\mbox{,}600 \pi-10\mbox{,}218\mbox{,}700\mbox{,}800 \bigr) \bigl(5 \pi^{10}-558 \pi^{8}+12\mbox{,}480 \pi^{6}-177\mbox{,}120 \pi^{4} \\ &{}+1\mbox{,}756\mbox{,}800 \pi^{2}-7\mbox{,}257\mbox{,}600\bigr) \bigl(3 \pi^{10}-1100 \pi^{8}+166\mbox{,}320 \pi^{6}-11\mbox{,}975\mbox{,}040 \pi^{4} \\ &{}+365\mbox{,}904\mbox{,}000 \pi^{2}-2\mbox{,}594\mbox{,}592 \mbox{,}000\bigr)>0. \end{aligned} \end{aligned}$$

Note that $0< x^{i}<(\frac{\pi}{2})^{i}, i=2,3, \forall x \in (0,\pi/2)$, we have $H_{2}(x) \leq (\rho_{2,0}+\rho_{2,2}\cdot (\frac{\pi }{2})^{2}+\rho_{2,3}\cdot (\frac{\pi}{2})^{3}) + \rho_{2,1} x + (\rho_{2,4}+\rho_{2,6}\cdot (\frac{\pi}{2})^{2}+\rho_{2,7}\cdot (\frac{\pi}{2})^{3}) x^{4} + \rho_{2,5} x^{5}+ (\rho_{2,8}+\rho_{2,10}\cdot (\frac{\pi}{2})^{2}+\rho_{2,11}\cdot (\frac{\pi }{2})^{3}) x^{8} + \rho_{2,9} x^{9} + (\rho_{2,12}+\rho_{2,14}\cdot (\frac{\pi }{2})^{2}) x^{12} + \rho_{2,13} x^{13} \approx -1.6 \cdot 10^{6} x^{13}-1.2 \cdot 10^{7} x^{12}-2.7 \cdot 10^{10} x^{9}-1.4 \cdot 10^{11} x^{8}-7.2 \cdot 10^{-13} x^{5}-3.7 \cdot 10^{14} x^{4}-2.5 \cdot 10^{16} x-1.1 \cdot 10^{17} < 0, \forall x \in (0,\pi/2)$. So we have $\Delta_{5}(x) \leq 0$ and $F(x) \leq R(x)$, $\forall x \in [0, \pi/2]$.

From the above discussions, we have completed the proof. □

## Discussions and conclusions

In principle, one can prove that $L_{i}(x) \leq L(x) \leq F(x) \leq R(x) \leq R_{i}(x)$, $\forall x \in [0,\pi/2]$ in a similar way, where $L_{i}(x)$ and $R_{i}(x)$, $i=2,3$, are two bounding functions in Eq. (6) and Eq. (7), respectively. The maximum errors between $F(x)$ and its different bounds are listed in Table 1. It shows that the bounds in this paper achieve a much better approximation than those of the bounds in Eq. (6) and Eq. (7).

**Table 1:** Maximum errors between $F(x)$ and its different bounds

Bounds	$L_{1}(x)$	$R_{1}(x)$	$L_{2}(x)$	$R_{2}(x)$	*L*(*x*)	*R*(*x*)
Error	2.8e−2	2.5e−4	2.09e−3	1.7e−3	1.37e−5	4.89e−6

The new method can be applied to refine the Becker–Stark inequality, which is studied in [5, 16, 24] and is known as

20 $$ \frac{8}{\pi^{2}-4 x^{2}} < \frac{\tan(x)}{x} < \frac{\pi^{2}}{\pi^{2}-4 x^{2}}, \quad \forall x \in (0,\pi/2). $$

Zhu [24] refined it as

21 $$ \begin{aligned}[b] \alpha_{l}(x) &= \frac{8}{\pi^{2}-4 x^{2}}+ \frac{2}{\pi^{2}}- \frac{(\pi^{2}-9)}{ 6 \pi^{4}} \cdot \bigl(\pi^{2}-4 x^{2} \bigr) < \frac{\tan(x)}{x} \\ & < \frac{8}{\pi^{2}-4 x^{2}}+ \frac{2}{\pi^{2}} - \frac{(10-\pi^{2})}{\pi^{4}} \cdot \bigl( \pi^{2}-4 x^{2} \bigr) =\alpha_{r}(x),\quad \forall x \in \biggl(0,\frac{\pi}{2} \biggr), \end{aligned} $$

while it is refined in [16] as follows:

22 $$ \begin{aligned}[b] \alpha_{2l}(x) &= \frac{8 +\mu(x)}{\pi^{2}-4 x^{2}} < \frac{\tan(x)}{x} \\ & < \frac{8 +\mu(x)+(\frac{32}{\pi^{3}}-\frac{8}{3 \pi})(\frac{\pi}{2}-x)^{3}}{\pi^{2}-4 x^{2}} = \alpha_{2r}(x), \quad \forall x \in \biggl(0, \frac{\pi}{2} \biggr), \end{aligned} $$

where $\mu(x)=\frac{8}{\pi}(\frac{\pi}{2}-x) + (\frac{16}{\pi^{2}}-\frac{8}{3})(\frac{\pi}{2}-x)^{2}$.

By applying the method in Sect. 2 and using the form $\frac{\sum^{6}_{i=0} \nu_{i} x^{i}}{\pi^{2}-4 x^{2}}$, one obtains the resulting bounds, $\beta_{l}(x)= \frac{\kappa_{1}(x)}{45 \pi^{6} (\pi^{2}-4 x^{2})}$ and $\beta_{r}(x)= \frac{\kappa_{2}(x)}{3 \pi^{6} (\pi^{2}-4 x^{2})}$, where $\kappa_{1}(x)=45 \pi^{8}+(-2 \pi^{8} -3660 \pi^{6} +36\mbox{,}000 \pi^{4}) x^{2}+(16 \pi^{7} +21\mbox{,}000 \pi^{5} -208\mbox{,}800 \pi^{3}) x^{3} +(-48 \pi^{6} -49\mbox{,}440 \pi^{4} +492\mbox{,}480 \pi^{2}) x^{4}+(64 \pi^{5} +54\mbox{,}240 \pi^{3} -541\mbox{,}440 \pi) x^{5} +(-32 \pi^{4} -23\mbox{,}040 \pi^{2} +230\mbox{,}400) x^{6}$ and $\kappa_{2}(x)=3 \pi^{8}+(-12 \pi^{6} +\pi^{8}) x^{2}+(5280 \pi^{3} -456 \pi^{5} -8 \pi^{7} x^{3})+ (-24\mbox{,}768 \pi^{2} +2272 \pi^{4} +24 \pi^{6}) x^{4}+(40\mbox{,}704 \pi -3808 \pi^{3} -32 \pi^{5}) x^{5}+(-23\mbox{,}040 +2176 \pi^{2} +16 \pi^{4}) x^{6}$, such that

$$\beta_{l}(x) < \frac{\tan(x)}{x} < \beta_{r}(x), \quad \forall x \in \biggl(0,\frac{\pi}{2} \biggr). $$

By using the Maple software, $\forall x \in (0,\frac{\pi}{2})$, it can be verified that $\beta_{l}(x)-\alpha_{l}(x)=-\frac{(\pi-2x)^{3}}{90 \pi^{6}}\times(57\mbox{,}600 x^{3}-8 \pi^{4} x^{3}-5760 \pi^{2} x^{3}+4920 \pi^{3} x^{2}-48\mbox{,}960 \pi x^{2}+ 4 \pi^{5} x^{2}+6210 \pi^{2} x-630 \pi^{4} x-105 \pi^{5}+1035 \pi^{3}) \approx -\frac{(\pi-2x)^{3}}{90 \pi^{6}} (-28.1940986 x^{3}-37.4163 x^{2}-77.48403 x-40.57055)>0$, $\beta_{r}(x) - \alpha_{r}(x)=\frac{1}{3 \pi^{6}} (\pi-2 x)^{2} x^{2} (-5760 x^{2}+544 \pi^{2} x^{2}+4 \pi^{4} x^{2}+4416 \pi x-408 \pi^{3} x-4 \pi^{5} x- 216 \pi^{2}+12 \pi^{4}+\pi^{6}) \approx \frac{1}{3 \pi^{6}} (\pi-2 x)^{2} x^{2} (-1.298840 x^{2}-1.36647 x-1.5362637)<0$, $\beta_{l}(x)-\alpha_{2l}(x)=-\frac{(\pi-2x)^{3}}{45 \pi^{6}}(28\mbox{,}800 x^{3}-4 \pi^{4} x^{3}-2880 \pi^{2} x^{3}-24\mbox{,}480 \pi x^{2}+2 \pi^{5} x^{2}+ 2460 \pi^{3} x^{2}+3240 \pi^{2} x-330 \pi^{4} x-75 \pi^{5}+720 \pi^{3}) \approx -\frac{(\pi-2x)^{3}}{45 \pi^{6}}(-14.0970443 x^{3}-18.7081400 x^{2}-167.48179 x- 626.95716)>0$ and $\beta_{l}(x)-\alpha_{2r}(x)=\frac{(\pi-2x)^{4}}{3 \pi^{6}}(\pi^{4} x^{2}-1440 x^{2}+136 \pi^{2} x^{2}-336 \pi x+34 \pi^{3} x-60 \pi^{2}+6 \pi^{4}) \approx \frac{(\pi-2x)^{4}}{3 \pi^{6}} (-0.324710 x^{2}-1.361725 x-7.7217177)<0$. So the bounds $\beta_{l}(x)$ and $\beta_{r}(x)$ achieve a better approximation than those results in both [24] and [16].
